# Beneficial effects of an investigational wristband containing Synsepalum dulcificum (miracle fruit) seed oil on the performance of hand and finger motor skills in healthy subjects: A randomized controlled preliminary study

**DOI:** 10.1002/ptr.5980

**Published:** 2017-11-23

**Authors:** Steven Gorin, Charles Wakeford, Guodong Zhang, Elvira Sukamtoh, Charles Joseph Matteliano, Alfred Earl Finch

**Affiliations:** ^1^ Institute of Sports Medicine and Orthopaedics Aventura Hospital Aventura FL 33180 USA; ^2^ Triangle Biostatistics Wake Forest NC 27587 USA; ^3^ Department of Food Science University of Massachusetts Amherst Amherst MA 01003 USA; ^4^ TEQ Solutions Kenmore NY 14217 USA; ^5^ Department of Kinesiology, Recreation and Sport Indiana State University Terre Haute IN 47809 USA

**Keywords:** elastomeric MFSO wristband, hand and finger dexterity, miracle fruit seed oil, motor skill performance, randomized controlled study, Synsepalum dulcificum

## Abstract

Miracle fruit (Synsepalum dulcificum) seed oil (MFSO) contains phytochemicals and nutrients reported to affect musculoskeletal performance. The purpose of this study was to assess the safety and efficacy of a compression wristband containing MFSO on its ability to measurably improve the hand and finger motor skills of participants. Healthy right‐handed participants (n = 38) were randomized in this double‐blind, placebo‐controlled study of MFSO and vehicle wristbands. Subjects wore the wristband on their left hand 4–6 weeks and then only on their right hand 2–4 weeks; the contralateral untreated hand served as an additional control. Twelve hand/finger motor skills were measured using quantitative bio‐instrumentation tests, and subject self‐assessment questionnaires were conducted. With each hand, in 9/12 tests, the MFSO group showed a clinically meaningful average improvement compared with an average worsening in the vehicle group. Statistical superiority to the control treatment group was exhibited in 9/12 tests for each hand (p < .01). After discontinuing the MFSO wristband on the left hand, test values regressed toward baseline levels. Subjects favored the MFSO wristband over the control, rating it as effective in improving their motor skills. Use of the MFSO wristband may improve an individual's manual dexterity skills and ability to maintain this performance.

**Abbreviations**
MFSO: miracle fruit seed oil
FTT: finger tapping test
FTTEP: finger tapping test explosive power
FTTA: finger tapping test acceleration
FTTF: finger tapping test fatigue
PPT: Purdue Pegboard Test
HSTPT: Hand Steadiness Tracing Pattern Test
HW: handwriting
GS: grip strength
PS: pinch strength
GSF: grip strength fatigue
PSF: pinch strength fatigue
N: newtons
anova: analysis of variance.

## 1 INTRODUCTION

Vegetable oils from plants and fruit seeds have been used for cosmetic and medicinal purposes as part of human culture for millennia. The ancient Greeks rubbed olive oil on the skin as an ergogenic aid during athletic competition to reduce muscle fatigue and enhance a faster recovery (Nomikos, Nomikos, & Kores, 2010). Synsepalum dulcificum seed oil, commonly known as miracle fruit seed oil (MFSO), is a rare and exotic fruit oil derived from the seed of the miracle fruit berry (Guney & Nawar, 1977). The MFSO is an abundant source of phytochemicals and essential nutrients that are known to regulate the physiologic functions of cells (Inglett & Chen, 2011). At greater than 20% of its weight, the bioactive‐rich unsaponifiable lipid fraction of the MFSO is among the highest in content recorded for a crude fruit seed oil (Del Campo, Zhang, & Wakeford, forthcoming). This fraction contains a substantial amount of phytochemicals such as the polyphenols, triterpenes, and phytosterols that exhibit antiinflammatory, antioxidant, and regenerative activities (Wu et al., 2013; Thirupathi, Silveira, Nesi, & Pinho, 2017; Loizou, Lekakis, Chrousos, & Moutsatsou, 2010; Lee et al., 2014; Del Campo et al., forthcoming) and appear to be beneficial for enhancing physical performance (Cases et al., 2017; Davis, Carlstedt, Chen, Carmichael, & Murphy, 2010; Yarahmadi et al., 2014). Appreciable quantities of essential nutrients are also present within the MFSO such as linoleic acid, vitamin K, and elemental silicon (unpublished observations) that can affect locomotor activity (Cocchetto, Miller, Miller, & Bjornsson, 1985; Raygada, Cho, & Hilakivi‐Clarke, 1998) and musculoskeletal health and homeostasis (Rodella, Bonazza, Labanca, Lonati, & Rezzani, 2014). During pilot studies to determine the safety and potential benefits of MFSO on improving the attributes of hair and alleviating skin conditions involving the hands, a number of subjects anecdotally described their hands and fingers feeling more nimble (Del Campo et al., forthcoming).

Elastomer gels and sheets have a wide range of cosmetic topical targeted delivery applications. For example, they have proven effective in the prevention of hypertrophic scars when applied as an adhesive under occlusion on skin (Foo & Tristani‐Firouzi, 2011). When bioactive substances, such as vitamin E, are incorporated into the gels, their efficacy is enhanced possibly due to the occlusion that occurs with the application of modest pressures through compression that has been shown to provide a simple noninvasive method to enhance the skin permeability of substances (Palmieri, Gozzi, & Palmieri, 1995; Zhai & Maibach, 2002). Elastomer gels have also been incorporated into wearables, such as sleeves and gloves, for orthopedic use to provide musculoskeletal support. In sports medicine, wearable compression garments that provide mechanical support have been utilized to improve physical performance (Kemmler et al., 2009). Studies have documented the benefits of wearing compressive garments for muscle function, recovery postexercise, motor control, thermoregulation, warming up, and range of motion (De Glanville & Hamlin, 2012; Hsu et al., forthcoming).

Widely accepted testing methods to evaluate hand and finger function have been routinely applied in neuropsychology and orthopedic clinical studies (Amirjani, Ashworth, Olson, Morhart, & Chan, 2011; Vega, 1969). These bio‐instrumentation tests have been previously reviewed for reliability, accuracy, validity, maintainability, and standardized administration and scoring procedures; and the number of practice trials needed to control for learning effects have been established (Robinette, Ervin, & Zehner, 1987). At times, certain modifications have been implemented to optimize the testing procedures for clinical studies (Porter, 2003). These bio‐instrumentation tests are available for evaluating different motor skill categories. For example, the Finger Tapping Test (FTT) and Handwriting (HW) Speed Test measure motor speed and the ability to make repeated movements (Hubel, Reed, Yund, Herron, & Woods, 2013; Prunty, Barnett, Wilmut, & Plumb, 2013), the Purdue Pegboard Test (PPT) measures manual dexterity (Ruff & Parker, 1993), and the Hand Steadiness Tracing Pattern Test (HSTPT) measures the speed and ability to keep the hand steady using fine precision movements while tracing a pattern (Jacobson, Winter‐Roberts, & Gemmell, 1991). Commercial equipment is routinely used to accurately measure grip and finger pinch strength with fatigue (Bohannon, 2003).

The objective of this preliminary clinical study was to assess the safety and efficacy of MFSO combined with compression on its ability to measurably improve the physical performance skills of the hands and fingers of healthy right‐handed subjects. To achieve this objective, a novel wearable compression wristband was developed that would contain the MFSO within a flexible elastomeric gel. When worn with firm compression, the wristband would effectively and continuously deliver the MFSO directly to the target area.

## 2 MATERIALS AND METHODS

### 2.1 Participants

Main criteria for inclusion were (a) age range 21–66 years; (b) healthy volunteers; (c) written informed consent; (d) confirmed as right‐handed by responses to the Edinburg Handedness Inventory survey form; (e) right hand performing significantly better than the left hand on more than half of the tests, as assessed with the motor skills tests used for this study [percent (%) difference calculation]; and (f) willing to not significantly change their normal daily physical routines during the study. Among the exclusion criteria were (a) any musculoskeletal upper extremity symptom or condition in the past year; (b) use of any medicine or treatment that may significantly affect the study outcome in the past 3 months; (c) a history of any medical or surgical event or condition that may significantly affect the study outcome, including cardiovascular disease, metabolic, renal, hepatic, or musculoskeletal disorders; (d) use of any new upper extremity training methods during the study; (e) use of any new drugs or performance enhancing products during the study; (f) use of caffeine, tea, energy drinks, or supplements during the 48 hr before each study visit; (g) use of any other upper extremity wearable product during the study; and (h) participation in another clinical trial or the use of an investigational product in the past 60 days. All clinical study procedures were approved by the Aspire Institutional Review Board (reference number PRO007) for the protection of human subjects. Before any study procedures were performed, the experimental procedures and risks/benefits were discussed with the subjects, and written informed consent was obtained.

### 2.2 Study design and testing protocol

The preliminary study was conducted as a double‐blind, placebo‐controlled clinical trial. Participants were evaluated by staff on four visits during the study; at an initial screening visit (Visit 1), at baseline/pretreatment (Visit 2), after 4–6 weeks of treatment (Visit 3), and after 6–10 weeks of treatment at the end of the study (Visit 4). At Visit 1, subjects that met the entrance criteria were enrolled into the study. At visit 2, subjects that continued to meet the entrance criteria were randomized and subsequently treated. One group of subjects received the wristband containing MFSO, the second group, an identical wristband without MFSO (vehicle only).

Study participants and staff were blinded with regard to which participants were in Groups 1(*n* = 23) and 2 (*n* = 15). The subjects were instructed to (a) wear their wristband snugly on the wrist for at least 3 hr every day, not to exceed 8 hr a day, for the duration of the study; (b) immediately report the occurrence of any adverse event to the staff and temporarily discontinue the use of the wristband until reevaluated; (c) complete a diary log form to document compliance with the daily use of the study wristband product and to report all adverse events; (d) not use the wristband when bathing or sleeping; (e) properly clean and store the wristband when not in use; and (f) wear the wristband on one designated hand; only on the left hand for the initial 4–6 weeks of treatment. At Visit 3, participants were evaluated and told to discontinue the use of the wristband on their left hand and wear the wristband only on the right hand until their next visit. At Visit 4, participants were evaluated, and the wristband and diary log form were collected. During Visits 3 and 4 (the two treatment visits), (a) participant diary log forms were reviewed to document compliance with the use of the study product, the reporting of any adverse events, and other comments; (b) safety evaluations were conducted, and the subjects asked to report any adverse events; (c) any change in the use of the concomitant medications related to the treatment conditions were documented; and (d) wristbands were inspected for signs of use. During all study visits, the participants had their hands subjected to a battery of bio‐instrumentation tests to evaluate their hand and finger motor skills.

### 2.3 Investigational product

**Figure 1:**
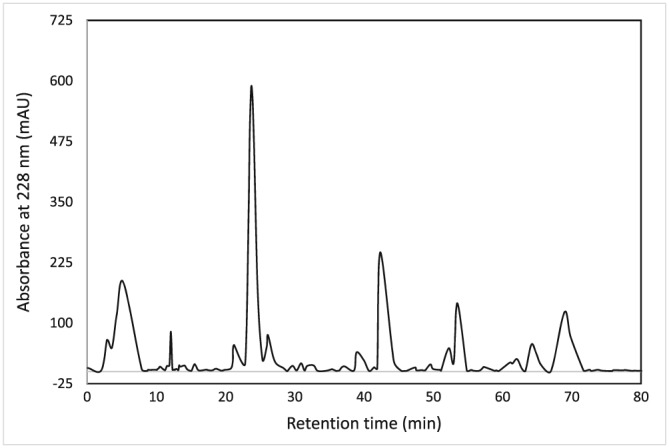
HPLC fingerprint of the total methanolic extract of the MFSO sample. A 100 mg/ml total methanolic extract of MFSO sample was analyzed using HPLC with detection wavelength at 228 nm. A combination of water and methanol with 0.1% acetic acid were used as mobile phases with the gradient of increasing methanol ratio over time.

The Synsepalum dulcificum seeds were secured from local growers in Africa, and the MFSO was extracted using supercritical CO_2_ fluid extraction methods in the USA (Pérez, Ruiz del Castillo, Gil, Blanch, & Flores, 2015). The yield of the crude oil extract was 8% (based on dry weight). The HPLC fingerprint of the total methanolic extract of the MFSO sample is shown in Figure 1. To improve the delivery of the MFSO, such that the MFSO could be released upon contact with the underlying skin progressively over time, the MFSO was incorporated within a flexible styrene‐ethylene/butylene‐styrene copolymer thermoplastic elastomeric gel. To confirm the proper amount of delivery over time, the exudation of the oil from the elastomer gelatinous composition to a surface was determined (Matteliano, Schaffer, & Sutton, 2010). These tests revealed that the gel was capable of releasing and properly delivering the oil for a period of months [unpublished observations]. To form the wristband and achieve targeted delivery of the MFSO, the elastomeric gel was heat bonded to an overlying stretchable fabric with one end attached to a Velcro strap. The wristbands containing MFSO (or vehicle control with no MFSO) were manufactured for the Miracle Fruit Oil Company (Miami Beach, FL, USA).The wristbands were the same size, and when worn to comfortably support the wrist without blocking movement, the average pressure generated was 29 mm ± 5 mm hg as per the manufacturer's instructions for comfortable fit. The wristband samples were received at the study site under code in blinded form and stored at ambient humidity and temperature.

### 2.4 Assessment of hand and finger motor skills

A battery of six characteristic and reliable quantitative hand and finger bio‐instrumentation tests used routinely in research studies and clinical practice were utilized to assess the effects of a MFSO wristband on the subject's motor skills performance (Table 1). Certain modifications from the accepted test methods were performed to assess new parameters or to optimize the measurement of the variables of interest. The tests quantitatively measured 12 hand and finger variables, which included finger tapping speed, endurance until fatigue, explosive power or quickness, and acceleration; accuracy and manual dexterity with fine and gross motor skills, hand steadiness and precision with errors, handwriting speed and mobility, and grip/pinch strength with fatigue. For each participant‐visit‐hand combination (e.g., Subject 1, Visit 2, left hand), each test was conducted twice in a test–retest format (i.e., replicate measurements). Specific clinical procedures were implemented for the performance of the bio‐instrumentation tests in an attempt to reduce procedural and interviewer test bias (Table 2).

**Table 1:** Motor skills assessments

Test	Assessment
Finger Tapping	Speed: Number of taps in 120 s. Fatigue: Number of taps at 10–30 s versus at 90–110 s Explosive power: Number of taps in 1st second. Acceleration: Time from 0–60 taps
Purdue Pegboard	Time to place and remove pins on a pegboard
Hand Steadiness Tracing Pattern	Time to trace complex segmented pattern. Number of errors
Handwriting	Number of letters written in 60 s
Grip Strength & Fatigue	Strength: Max force at 0–10 s. Fatigue: Max force at 0–10 s versus at 90–100 s
Pinch Strength & Fatigue	Strength: Max force at 0–10 s. Fatigue: Max force at 0–10 s versus at 50–60 s

**Table 2:** Clinical procedures for motor skills assessments

(a) All testing was performed in a quiet, isolated, and enclosed room to reduce the effects of outside visual and auditory interference.
(b) Subjects were evaluated in the same room with the same lighting, same chair and desk, and instructed to maintain the same posture for the performance of each test to reduce the effects of the room environment.
(c) Subjects were tested at the same time period on every visit (either morning or afternoon) to reduce the effects due to the time of day.
(d) Subjects were only allowed to wear the wristband (no jewelry, watches, or other garments worn on their upper extremities), and mobile phones were shut off during the performance of the tests.
(e) An investigator‐generated randomization procedure was used to reduce the effect of the test order.
(f) Subjects were assigned to and evaluated by the same staff investigator during all of their visits.
(g) Standardized written instructions were given to each participant for them to read to familiarize themselves with the testing procedures.
(h) The investigator provided the same verbal instructions in the same tone of voice during each test to reduce the effects due to coaching.
(i) Subjects were instructed to alternate their sequence of which hand to use first among each different test to reduce the effect of hand order.
(j) Subjects performed each test alternating each hand in duplicate studies (total of four independent runs for each test during each visit).
(k) Subjects were provided with two practice runs for each test (one run with the use of each hand) at the screening visit to become familiarized with the performance of each test.
(l) Subjects were instructed to have a warm‐up practice period (3–5 min) prior to the official performance of their first test of the baseline and posttreatment visits to reduce a lack of preparedness.
(m) Subjects had to complete each individual test without a break (four independent runs) but were allowed up to a 5‐min break to reduce fatigue before proceeding to a different test.
(n) Test results were recorded by staff members in the subject case report forms immediately after each test was completed.
(o) Subjects performed and completed all tests in 1 day during each study visit.

On each visit, all subjects' hand and finger motor skill performances were assessed with the same battery of bio‐instrumentation tests. For all tests, the percent difference between right and left hand performance (mean and max values) was calculated and used to determine if the subject met the relevant entrance criterion.

*FTT with fatigue (FTTF)*: The FTT was used to measure finger tapping movements and was performed using a digital FTT App designed by Sybu Data (Pty) LTD (Cape Town, South Africa; www.sybu.co.za). The FTT was modified to include an extended 120‐s duration (normally at 10 s) to allow for the measurement of finger fatigue. The speed of finger tapping was measured for the right and left index finger separately as described (Vega, 1969). The results were expressed as the number of taps for each individual run including the maximal and mean values among the replicate tests for each hand. It was arbitrarily decided to select and compare a 20‐s time interval near the beginning (at the 10‐ to 30‐s interval) and end of the test (at the 90‐ to 110‐s interval) for the measurements of finger fatigue. The percentage of FTTF value for each run was calculated as the difference between the FTT number of taps among the two intervals divided by the FTT number of taps during the first interval × 100. The FTT explosive power (FTTEP) and FTT acceleration (FTTA) were defined as the number of taps during the first second and the time (seconds) needed to reach 60 taps, respectively.

*PPT:* Fine finger dexterity and gross hand movements were measured using the PPT as described by the manufacturer (Lafayette Instrument Company, Lafayette, IN, 47904, USA). The PPT was modified such that the new endpoint was the time (seconds) needed to complete the placement of all 50 pins into the pegboard and placed back into the cups of origin.

*HSTPT:* Hand steadiness using the HSTPT to measure fine precision movements was performed as previously described (Robinette et al., 1987). The test was scored by the duration of time (seconds) it took the subject to complete all the segments in the pattern. If the participant raised the pen, moved the paper (more than ½ inch in any direction at any given time), crossed a line within a segment, or if the crossed line spanned additional adjacent segments, it was counted as an error. Subjects were allowed up to 5 errors with the right hand and 20 errors with the left for the test to be counted.

*HW Speed test:* Gross and fine single‐handed motor skills were measured using the HW Speed test as previously described (Prunty et al., 2013). The subject was instructed to write their first name on standard lined paper as many times as possible in 60 s. The test was scored based upon the total number of letters written.

*GS and GSF:* Maximal isometric and sustained grip force with fatigue were measured using a hand‐held dynamometer as per the manufacturer's instructions (Vernier Software and Technology, Beaverton, OR, 97005, USA). For the maximal GS value, the subject applied maximal GS pressure for 10 s. The percentage of difference in the maximal gripping force (and mean force) GS differentials between the subjects' right and left hands were calculated in newtons (N). For the sustained GS, the subject applied maximal pressure for 100 s. The percentage of GSF maximal value for each run was calculated as the difference between the GS max among the two intervals (during the 0‐ to 10‐s and 90‐ to 100‐s intervals) divided by the GS max during the first interval × 100.

*PS and PSF:* Maximal isometric and sustained pinch forces with fatigue were measured using the same equipment as for GS. For the maximal and sustained PS values, the participant applied maximal PS pressure for 10 and 60 s, respectively. The second maximum PS measurement was taken at the 50‐ to 60‐s interval. The data entry and calculations for PS and PSF were the same as with the GS and GSF.

### 2.5 Questionnaires

Participants completed a self‐assessment hand and finger performance outcome questionnaire at every visit. Participants rated the performance of their hands and fingers with regard to movements, strength, and sensation with the use of each hand using a 1 (*most positive*) to 5 (*most negative*) rating scale. At Visits 3 and 4, participants were also asked to document if a perceptual benefit in mobility, strength, or sensation occurred, compared with Visits 1 and 2 and if so, to describe the benefit in detail. They were also asked if they noticed improvements in accomplishing certain tasks with the use of both hands. At Visit 4, participants answered a product use assessment questionnaire providing their degree of satisfaction with the wristband product. They were also asked to reveal any perceptual improvements in mobility, skills, strength, and endurance as well as in their performance of 22 commonly routine specific tasks such as typing, texting, and writing.

### 2.6 Statistical analysis

The sample size for this preliminary study was selected to sufficiently characterize the performance of the MFSO treatment group and to provide adequate power to assess the amount of the MFSO group performance that is above and beyond that observed in the gel band control group. Sample sizes of at least 20 subjects in the MFSO group and at least 15 subjects in the control group were deemed to meet these criteria, according to the power analysis. For example, relative to the improvement in the number of finger taps (Finger Tapping Test), using a two‐sided independent sample *t*‐tests, a sample size of 20 in the MFSO group and 15 in the control group provided at least 90% power to detect a difference of 30 taps between treatment groups, assuming a common standard deviation of 25 taps and 0.05 level of significance.

For each hand test variable, the primary analysis was improvement from baseline (defined as Visits 3 and 2 for left hand and Visits 4 and 3 for right hand). A secondary analysis examined the change from baseline at Visit 4 for the left hand. The arithmetic mean of the two replicate measurements was used for the analysis. The two treatment groups were compared at baseline to assess homogeneity and at endpoint using a one‐way analysis of variance (anova) at the 0.05 level of significance. Questionnaire data were summarized by treatment group by presenting the number and percentage of subjects included in a given category. No statistical testing was performed on the questionnaire data. Bio‐instrumentation study data were analyzed using SAS® Software Version 9.2.

## 3 RESULTS

Forty‐six healthy right‐handed subjects were randomized and received treatment. Eight subjects voluntarily withdrew due to personal reasons unrelated to the treatment and did not complete the study, resulting in a total of 38 participants completing the study (MFSO, *n* = 23; Vehicle, *n* = 15). Subject ages ranged from 21 to 66 years with a mean (SD) of 42.8 (12.98) years. Most subjects were male (73.7%) and most had a college or postgraduate degree (57.9%). Self‐reported adherence to the study wristband among all subjects was equally high across the two treatment groups (>90% diary‐logged days of use). No subject reported any adverse reaction with the use of any of the wristbands.

Using a significance level of 0.05, none of the statistical tests for left hand assessments at baseline resulted in statistically significant differences between the two treatment groups. Three tests [PPT, HSTPT (errors), and GSF] for the right hand showed some departure from homogeneity at baseline, but in none of these cases was this departure considered clinically remarkable. Therefore, the assumption of homogeneity at baseline was reasonable, and the change from baseline for each variable was a meaningful endpoint or reference.

### 3.1 Effect of MFSO on hand and finger motor skills

**Figure 2:**
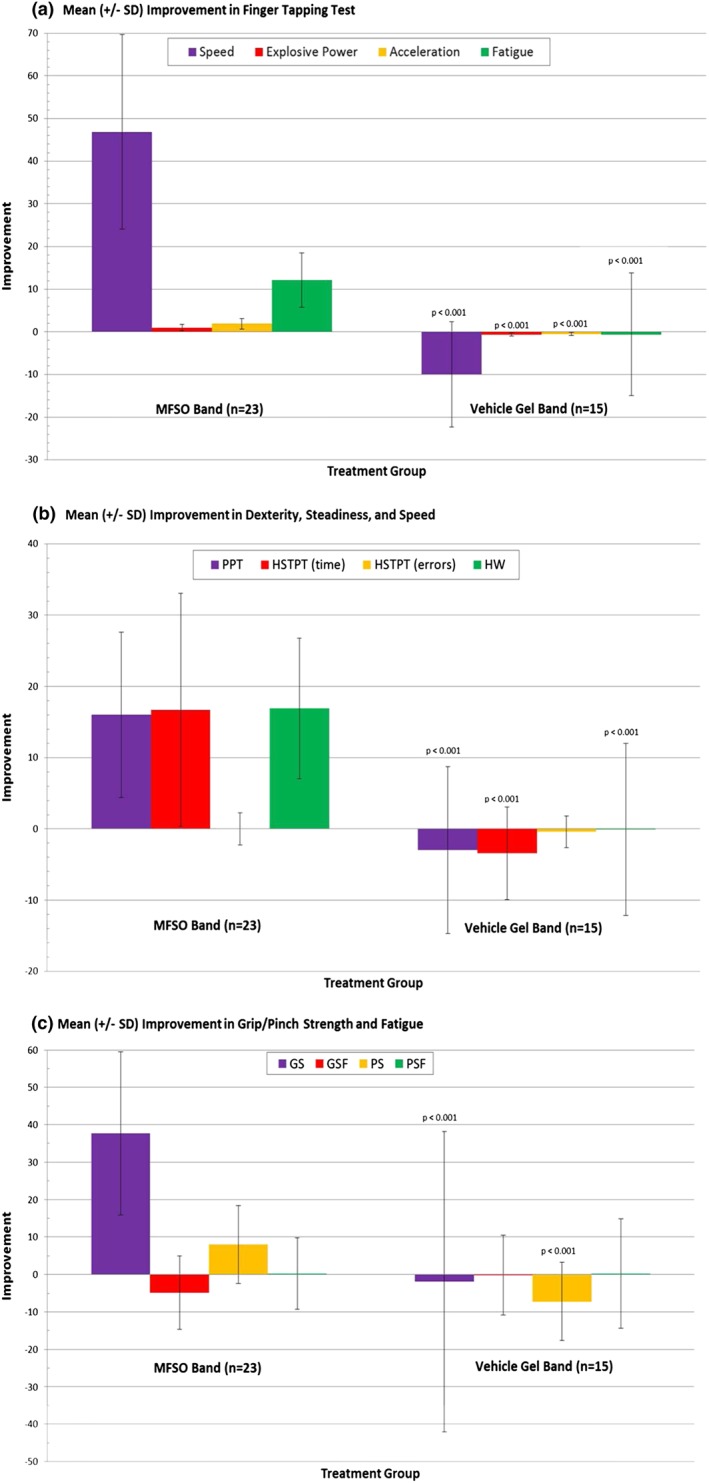
Left hand assessments by treatment group. Improvement (mean ± SD) from Visit 2 (baseline) at Visit 3 by treatment group in left hand (a) finger tapping tests, (b) dexterity (PPT), steadiness (HSTPT), and speed (HW speed test), and (c) grip/pinch strength and fatigue. For each test, the MFSO band treatment group was compared with the control group using a one‐way ANOVA. MFSO = miracle fruit seed oil; PPT = Purdue Pegboard Test; HSTPT = Hand Steadiness Tracing Pattern Test; HW = handwriting; GS = grip strength; PS = pinch strength; GSF = grip strength fatigue; PSF = pinch strength fatigue [Colour figure can be viewed at wileyonlinelibrary.com]

**Figure 3:**
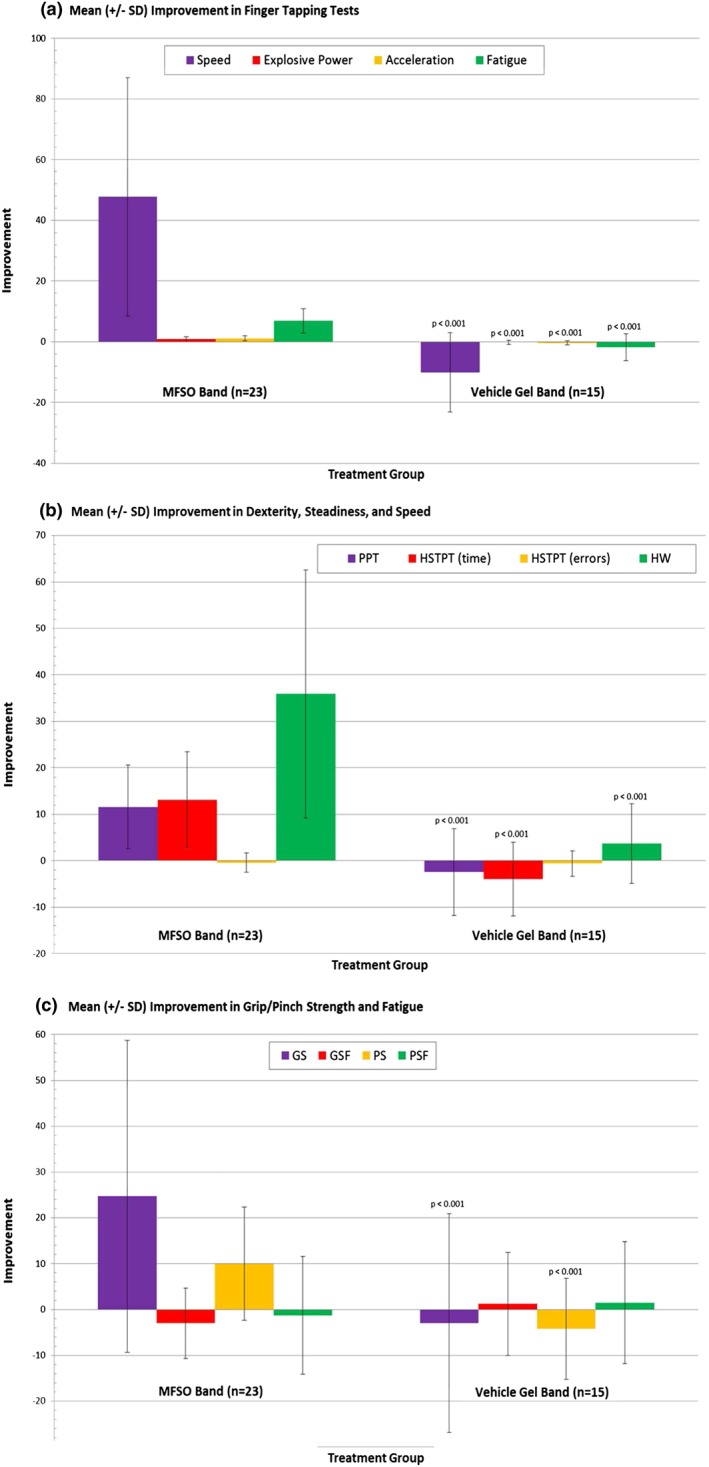
Right hand assessments by treatment group. Improvement (mean ± SD) from Visit 3 (baseline) at Visit 4 by treatment group in right hand (a) finger tapping tests, (b) dexterity (PPT), steadiness (HSTPT), and speed (HW speed test), and (c) grip/pinch strength and fatigue. For each test, the MFSO band treatment group was compared with the control group using a one‐way ANOVA. MFSO = miracle fruit seed oil; PPT = Purdue Pegboard Test; HSTPT = Hand Steadiness Tracing Pattern Test; HW = handwriting; GS = grip strength; PS = pinch strength; GSF = grip strength fatigue; PSF = pinch strength fatigue [Colour figure can be viewed at wileyonlinelibrary.com]

Mean improvements in the hand and finger motor skills parameters were evident with the use of the MFSO band during both treatment intervals; after 4–6 (Visit 3) and 2–4 weeks (Visit 4) of use on the left and right hand, respectively (Figures 2 and 3). With each hand, in 9 of 12 bio‐instrumentation tests, the MFSO group showed a clinically meaningful average improvement compared with an average worsening in the vehicle group (Tables 3 and 4). The average MFSO group improvement and difference between treatment group means of the left/right hands were FTT 46.9 taps, 56.8 taps (*p* < .0001)/47.7 taps, 57.8 taps (p < .0001); FTTEP 1.0 tap, 1.6 taps (p < .0001)/0.8 tap, 1.0 taps (*p* = .0004); FTTA 1.9 s, 2.4 s (p < .0001)/1.1 s, 1.5 s (p < .0001); FTTF 12.1%, 12.7% (*p* = .0007)/6.9%, 8.7% (p ˂ .0001); PPT 16 s, 19.0 s (p < .0001)/11.6 s, 14.0 s (p < .0001); HSTPT 16.7 s, 20.1 s (p < .0001)/13.1 s, 17.0 s (p < .0001); HW Speed test 16.9 letters, 17.0 letters (p < .0001)/35.9 letters, 32.2 letters (p < .0001); GS 37.7 N, 39.6 N (p = .0004)/24.7 N, 27.7 N (*p* = .0096); and PS 8.0 N, 15.2 N (p < .0001)/10.0 N, 14.2 N (*p* = .001). No differences were found in the HSTPT errors, GSF, and PSF tests. The placebo control treatment group did not perform statistically better than the MFSO band treatment group in any of the 12 hand and finger tests.

**Table 3:** Left hand assessments

Variable	Endpoint	MFSO band (n = 23)	Vehicle gel band (n = 15)	*p* value
1. Finger Tapping Tests: Speed, explosive power, acceleration, and fatigue				
Speed (# of taps)	Baseline (Visit 2) mean (SD)	496.7 (66.30)	511.2 (44.36)	.4594
	Improvement from baseline at Visit 3 mean (SD)	46.9 (22.85)	−9.9 (12.34)	<.0001
	Improvement from baseline at Visit 4 mean (SD)	17.1 (48.97)	−18.9 (17.38)	NT
Explosive power (# taps in the first second)	Baseline (Visit 2) mean (SD)	4.7 (0.79)	5.0 (0.53)	.1439
	Improvement from baseline at Visit 3 mean (SD)	1.0 (0.73)	−0.6 (0.39)	<.0001
	Improvement from baseline at Visit 4 mean (SD)	0.5 (0.80)	−0.4 (0.44)	NT
Acceleration [time to 60 taps (seconds)]	Baseline (visit 2) mean (SD)	13.1 (2.37)	12.3 (0.99)	.2613
	Improvement from baseline at Visit 3 mean (SD)	1.9 (1.26)	−0.5 (0.38)	<.0001
	Improvement from baseline at Visit 4 mean (SD)	0.6 (1.86)	−0.7 (0.73)	NT
Fatigue (%)	Baseline (visit 2) mean (SD)	13.5 (6.54)	13.0 (12.64)	.8788
	Improvement from baseline at Visit 3 mean (SD)	12.1 (6.39)	−0.6 (14.41)	.0007
	Improvement from baseline at Visit 4 mean (SD)	4.3 (7.93)	2.3 (13.41)	NT
2. Dexterity, steadiness, and speed				
Purdue Pegboard Test (seconds)	Baseline (Visit 2) mean (SD)	174.1 (14.98)	180.9 (24.97)	.2997
	Improvement from baseline at Visit 3 mean (SD)	16.0 (11.59)	−3.0 (11.69)	<.0001
	Improvement from baseline at Visit 4 mean (SD)	5.8 (12.28)	−2.2 (5.87)	NT
Hand Steadiness Tracing Pattern Test (seconds)	Baseline (Visit 2) mean (SD)	85.4 (21.86)	78.9 (19.70)	.3549
	Improvement from baseline at Visit 3 mean (SD)	16.7 (16.40)	−3.4 (6.50)	<.0001
	Improvement from baseline at Visit 4 mean (SD)	5.7 (15.41)	−4.9 (6.39)	NT
Hand Steadiness Tracing Pattern Test (# of errors)	Baseline (Visit 2) mean (SD)	4.5 (2.88)	6.5 (3.95)	.0793
	Improvement from baseline at Visit 3 mean (SD)	0.0 (2.27)	−0.4 (2.24)	.5279
	Improvement from baseline at Visit 4 mean (SD)	−0.7 (2.63)	−2.0 (3.46)	NT
Handwriting Speed Test (# of letters)	Baseline (Visit 2) mean (SD)	70.0 (20.80)	64.9 (30.95)	.5473
	Improvement from baseline at Visit 3 mean (SD)	16.9 (9.87)	−0.1 (12.06)	<.0001
	Improvement from baseline at Visit 4 mean (SD)	11.3 (17.29)	−3.7 (13.15)	NT
3. Grip/pinch strength and fatigue				
Grip strength maximum force (N)	Baseline (Visit 2) mean (SD)	212.8 (85.99)	196.7 (72.60)	.5544
	Improvement from baseline at Visit 3 mean (SD)	37.7 (21.79)	−1.9 (40.13)	.0004
	Improvement from baseline at Visit 4 mean (SD)	5.4 (41.17)	−5.5 (49.01)	NT
Grip strength fatigue (%)	Baseline (Visit 2) mean (SD)	64.0 (8.52)	66.0 (5.61)	.4224
	Improvement from baseline at Visit 3 mean (SD)	−4.9 (9.84)	−0.2 (10.65)	.1768
	Improvement from baseline at Visit 4 mean (SD)	−0.5 (8.94)	−0.7 (8.37)	NT
Pinch strength maximum force (N)	Baseline (Visit 2) mean (SD)	68.7 (20.56)	70.3 (20.01)	.8147
	Improvement from baseline at Visit 3 mean (SD)	8.0 (10.36)	−7.2 (10.45)	<.0001
	Improvement from baseline at Visit 4 mean (SD)	2.1 (20.35)	−10.1 (16.53)	NT
Pinch strength fatigue (%)	Baseline (visit 2) mean (SD)	59.4 (7.35)	57.6 (14.42)	.6039
	Improvement from baseline at Visit 3 mean (SD)	0.2 (9.53)	0.2 (14.65)	.9970
	Improvement from baseline at Visit 4 mean (SD)	−0.3 (10.52)	3.9 (16.98)	NT

*Note*. *p* value is from a one‐way anova *F*‐test. MFSO = miracle fruit seed oil; SD = standard deviation; NT = not tested; N = newtons.

**Table 4:** Right hand assessments

Variable	Endpoint	MFSO band (n = 23)	Vehicle gel band (n = 15)	*p* value
1. Finger tapping tests: Speed, explosive power, acceleration, and fatigue				
Speed (# of taps)	Baseline (Visit 3) mean (SD)	559.8 (58.36)	563.0 (53.00)	.8634
	Improvement from baseline at Visit 4 mean (SD)	47.7 (39.25)	−10.1 (13.10)	<.0001
Explosive power (# taps in the first second)	Baseline (Visit 3) mean (SD)	5.2 (0.84)	5.1 (0.74)	.6934
	Improvement from baseline at Visit 4 mean (SD)	0.8 (0.89)	−0.2 (0.62)	.0004
Acceleration [time to 60 taps (seconds)]	Baseline (Visit 3) mean (SD)	11.7 (1.42)	11.9 (1.29)	.5797
	Improvement from baseline at Visit 4 mean (SD)	1.1 (0.81)	−0.4 (0.66)	<.0001
Fatigue (%)	Baseline (Visit 3) mean (SD)	8.5 (3.08)	8.1 (3.65)	.7165
	Improvement from baseline at visit 4 mean (SD)	6.9 (4.01)	−1.8 (4.42)	<.0001
2. Dexterity, steadiness, and speed				
Purdue Pegboard Test (seconds)	Baseline (Visit 3) mean (SD)	152.3 (9.86)	165.3 (20.16)	.0120
	Improvement from baseline at Visit 4 mean (SD)	11.6 (9.01)	−2.4 (9.32)	<.0001
Hand Steadiness Tracing Pattern Test (seconds)	Baseline (Visit 3) mean (SD)	63.5 (17.39)	63.2 (18.89)	.9617
	Improvement from baseline at Visit 4 mean (SD)	13.1 (10.32)	−3.9 (7.92)	<.0001
Hand Steadiness Tracing Pattern Test (# of errors)	Baseline (Visit 3) mean (SD)	2.1 (1.64)	3.8 (2.23)	.0117
	Improvement from baseline at Visit 4 mean (SD)	−0.4 (2.12)	−0.6 (2.70)	.8017
Handwriting Speed Test (# of letters)	Baseline (Visit 3) mean (SD)	162.9 (27.31)	152.2 (39.35)	.3252
	Improvement from baseline at Visit 4 mean (SD)	35.9 (26.70)	3.7 (8.61)	<.0001
3. Grip/pinch strength and fatigue				
Grip strength maximum force (N)	Baseline (Visit 3) mean (SD)	238.3 (87.37)	210.1 (70.85)	.3031
	Improvement from baseline at Visit 4 mean (SD)	24.7 (34.04)	−3.0 (23.86)	.0096
Grip strength fatigue (%)	Baseline (Visit 3) mean (SD)	62.8 (9.33)	69.0 (6.66)	.0325
	Improvement from baseline at Visit 4 mean (SD)	−3.0 (7.66)	1.2 (11.26)	.1791
Pinch strength maximum force (N)	Baseline (Visit 3) mean (SD)	72.5 (21.00)	76.3 (23.30)	.6036
	Improvement from baseline at Visit 4 mean (SD)	10.0 (12.38)	−4.2 (11.01)	.001
Pinch strength fatigue (%)	Baseline (Visit 3) mean (SD)	54.8 (11.20)	59.3 (10.89)	.2274
	Improvement from baseline at Visit 4 mean (SD)	−1.3 (12.86)	1.5 (13.33)	.5236

*Note*. *p* value is from a one‐way anova *F*‐test. MFSO = miracle fruit seed oil; SD = standard deviation; N = newtons.

The hand outcomes and wristband use questionnaires analyses indicated that the use of the MFSO wristband was associated with a greater level of favorable responses when compared with the control group. At Visit 3, more than 50% of the subjects in the MFSO band treatment group reported an improvement in left hand strength and in accomplishing daily tasks compared with less than 22% in the control group. At Visit 4, a similar response occurred with the right hand outcomes. For each of the four categories (i.e., movement, skill, strength, and endurance), at least 39% of the subjects in the MFSO band treatment group reported an improvement using the wristband compared with less than 14% in the control group. In all but one of the 18 improvement categories, the percentage of subjects reporting improvement in the MFSO band treatment group was more than twice that of the control treatment group.

### 3.2 Effect during no treatment on hand and finger motor skills

**Figure 4:**
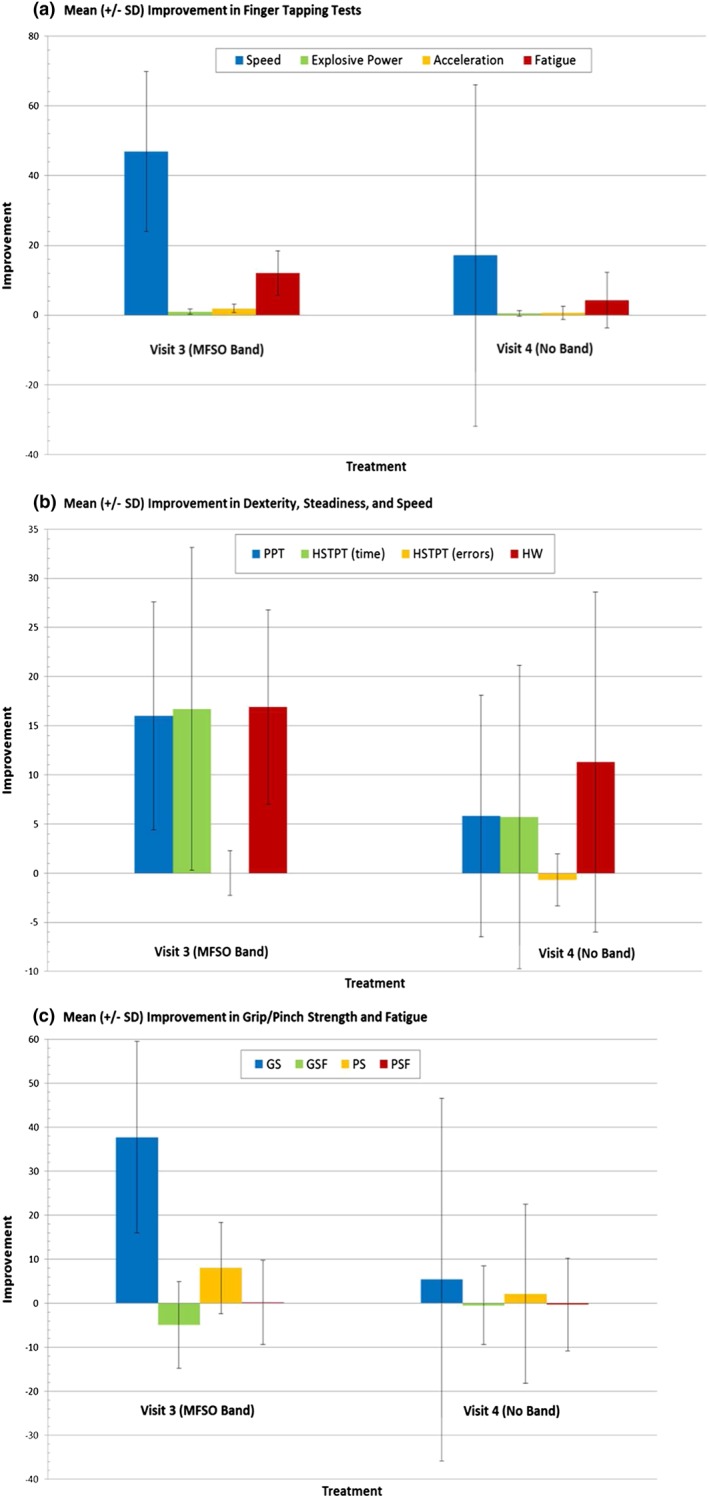
Left hand assessments: MFSO group with and without band. Improvement (mean ± SD) from Visit 2 (baseline) at Visit 3 (with band) and at Visit 4 (without band) for the MFSO band treatment group in left hand (a) finger tapping tests, (b) dexterity (PPT), steadiness (HSTPT), and speed (HW speed test), and (c) grip/pinch strength and fatigue. MFSO = miracle fruit seed oil; PPT = Purdue Pegboard Test; HSTPT = Hand Steadiness Tracing Pattern Test; HW = handwriting; GS = grip strength; PS = pinch strength; GSF = grip strength fatigue; PSF = pinch strength fatigue [Colour figure can be viewed at wileyonlinelibrary.com]

After 4–6 weeks of no treatment with the right‐hand (Visit 3), there were no clinically meaningful changes (from Visits 2 to 3) for any of the 12 right hand tests (data not shown). After 2–4 weeks of no treatment with the left hand (Visit 4), there was a noticeable return toward baseline (Visit 2) levels in all nine left hand tests that had previously shown an improvement (Figure 4). The decline was not extreme, and for the FTT, FTTF, PPT, HSTPT (seconds), and HW speed test, a modest portion of the effect observed at Visit 3 was preserved at Visit 4.

## 4 DISCUSSION

This study is the first clinical trial to demonstrate the safety and efficacy of the use of a fruit seed oil incorporated into a wearable compression garment for improving the performance of hand and finger motor skills. This was also the first placebo‐controlled, double‐blind, clinical study that compared the efficacy of this unique type of combination product with its vehicle. The results of this preliminary clinical study demonstrated a meaningful within MFSO band treatment group improvement in 9 of 12 bioinstrumentation motor skills tests with the use of either hand in right‐handed subjects. In addition, the subjects in the MFSO band treatment group robustly demonstrated statistically significant and clinically meaningful improvement compared with the placebo control group with respect to finger tapping measurements, hand dexterity metrics, and strength assessments. The differences between treatment means were quite substantial that would represent a marked performance benefit when using the MFSO band compared with the control treatment. Furthermore, the self‐assessments also demonstrated that subjects favored the MFSO band over the control band on all tested attributes.

In all control participants, there were (a) no instances where the control group was statistically superior to the MFSO band with regard to improvement; (b) numerous instances where the control group led to a decline in performance skills or an insignificant improvement; and (c) numerous instances where the improvement in the MFSO band was statistically superior to the improvement seen with the control group. These findings suggest that compression is not sufficient to provide the beneficial results that were observed. A limitation of the study design was that it did not address the use of the MFSO alone without the compression wristband. It is possible that MFSO or other vegetable oils could exert a beneficial effect on hand performance after their direct application on the wrist independent of the use of compression. However, the messy and greasy application of oils and the need for repeated applications would be expected to reduce compliance. In addition, if the full benefit in improvement of performance can only be realized with the use of the oil combined with a durable garment that provides compression, the wristband provides an efficient and practical choice for enhanced subject compliance.

This study design approach provided added benefits because it allowed for a comparison with the contralateral hand serving as an additional no band treatment control and an evaluation of efficacy at two different treatment time intervals. During the use of the MFSO band exclusively on the left hand for 4–6 weeks, the subject's untreated right hand values remained near baseline and showed no clinically meaningful improvements in performance skills. The switch of the MFSO band to its exclusive use on the right hand for 2–4 weeks resulted in an improvement of the right hand performance skills and a concomitant reduction in the performance skills of the left hand. The left hand manifested a major decline returning in the direction toward near‐baseline levels of performance in nearly all of the tests, reversing most of the gains in the improvements previously obtained with the use of the MFSO wristband. These results indicate that for the favorable benefits to continue and persist over time, the MFSO wristband should be routinely worn and not discontinued. This finding also adds further support to the original hypothesis that the beneficial results in hand and finger performance skills that were observed are associated with the exclusive wear of the MFSO wristband. The degree of improvement in physical performance skills was fairly similar when wearing the MFSO band on the left or right hand, indicating that the beneficial effects on performance occurred as early as after 2–4 weeks of treatment.

Apart from this randomized study, 10 additional subjects who did not participate in the study, but had similar entry criteria, agreed to be simultaneously followed and assessed with the use of the commercially available Power Balance® band (*n* = 4; Verdan et al., 2012) and on no treatment (*n* = 6) according to the study timelines. This exploratory analysis was conducted to observe (a) the effects of a popular commercial brand of performance wristband and (b) the typical variability in hand/finger motor skills over time in subjects with no band. In the four subjects that wore the Power Balance® band, there was a tendency toward a negative mean improvement from baseline values in the majority of the tests that were performed for both hands, possibly indicating that hand performance skills may worsen with the use of certain bands. In the six subjects that received no treatment, the changes in the motor skills tests were unremarkable.

Although the exact mechanism of action for the enhanced performance effects observed with the MFSO band remains unknown, it can be speculated that the MFSO band improves the stability, mobility, and flexibility of the wrist joint. The combination of sustained and prolonged firm mechanical compression, the occlusion formed upon contact of the gel with skin, the lubricity imparted by the oil, and the ingredients in the MFSO may play a role. Prolonged compression and occlusion of the skin with a highly lubricated oil‐based elastomeric gel may produce slight increases in the local temperature and regional circulation that could affect the functional mobility of the underlying tissues.

Apart from or in combination with the mechanical and physical effects, the phytochemicals and nutrients in the MFSO may have an important physiological role in the observed effect (Guney & Nawar, 1977; Inglett & Chen, 2011). Sufficient evidence exists in Ayurveda and traditional Chinese medicine for the support of the effectiveness of the topical application of herbal preps and bioactive oils, alone or in combination with physical modalities, acting as antioxidants and antiinflammatory agents for the management of musculoskeletal conditions, such as back pain, joint stiffness, and arthritis (Cibere et al., 2003; Shoara et al., 2015; Yip & Tse, 2004). Although the composition of the MFSO is complex and relatively unexplored, it contains abundant amounts of phytochemical compounds such as the polyphenols, triterpenes, and phytosterols that exhibit antioxidant, antiinflammatory, and regenerative activities (Del Campo et al., forthcoming; Wu et al., 2013; Thirupathi et al., 2017; Loizou et al., 2010; Lee et al., 2014). Polyphenols, such as the anthocyanins and flavonoids, appear to be beneficial for improving physical performance during exercise (Cases et al., 2017; Davis et al., 2010; Yarahmadi et al., 2014). In addition to acting as antioxidants in the prevention of exercise induced muscle damage, polyphenols affect endothelial cell nitric oxide synthetase leading to vasorelaxation and increases in blood flow to musculoskeletal tissues, thereby improving locomotion (Goldfarb, 1999; Lorenz et al., 2004). Musculoskeletal locomotor function and homeostasis may also be affected by the essential nutrients contained within the MFSO, such as vitamin K, linoleic acid, and elemental silicon (Rodella et al., 2014). For example, vitamin K deficiency (Cocchetto et al., 1985) has been reported to reduce locomotor activity whereas a high dietary intake of linoleic acid (Raygada et al., 1998) increased the activity, reducing the time spent immobile. Furthermore, in clinical studies, vitamin K and silicon supplementation were shown to improve bone health favoring regeneration while reducing resorption (Craciun, Wolf, Knapen, Brouns, & Vermeer, 1998; Spector et al., 2008). Nevertheless, the identification of the compound(s) contributing to the effect on the performance parameters and the mechanism of action need to be investigated and require further study.

The use of the MFSO band may also provide significant benefits to persons wanting an improvement when performing manual tasks to enhance productivity during activities of daily living. For example, a participant noted that while wearing the MFSO wristband, he was able to use his left hand much better to text on his phone and perform more push‐ups. Also, several subjects reported that their video‐gaming skills improved and were able to win more games due to faster hand and finger movements when handling the controller. Despite these favorable accounts, the full benefits in performance may not be realized if persons do not continue the use of the product for long enough. To maximize compliance with the use of the wristband, education will be needed to motivate users by encouraging confidence in its safety and benefits as well as providing reassurance with appropriate expectations. Innovative strategies focusing on the targeted topical delivery of phytochemicals and nutrients as ergogenic aids for improving functional performance and enhancing productivity represents an important avenue for future investigations.

## 5 CONCLUSIONS

Right‐handed subjects that used the MFSO band demonstrated clinically meaningful improvements when compared with the vehicle band control group with respect to finger tapping measurements, hand dexterity metrics, and strength assessments. When worn, a wristband containing MFSO can act as an ergogenic aid to improve an individual's hand and finger motor skills and ability to maintain this performance.

## CONFLICT OF INTEREST

S. G., G. Z., E. S., C. J. M., and A. E. F. have no conflict of interest. C. W. received consulting support from the Miracle Fruit Oil Company.
